# In-hospital rapid response system: effects on outcome and workload

**DOI:** 10.1186/cc11113

**Published:** 2012-03-20

**Authors:** D Liberti, C Di Maria, P De Luca, M Alberico, C Popa, O Sagliocco, MR Scalzulli, E De Blasio

**Affiliations:** 1 Hospital G. Rummo, Benevento, Italy

## Introduction

The implementation of an in-hospital rapid response system (RRS) could improve the outcome of a deteriorating patient but could increase the medical emergency team (MET) and ICU staff workload [1,2].

## Methods

A retrospective analysis of the years pre, during and post implementation of a RRS in a 480-bed hospital with a mean of 17,500 admissions/year.

## Results

**Figure 1:**
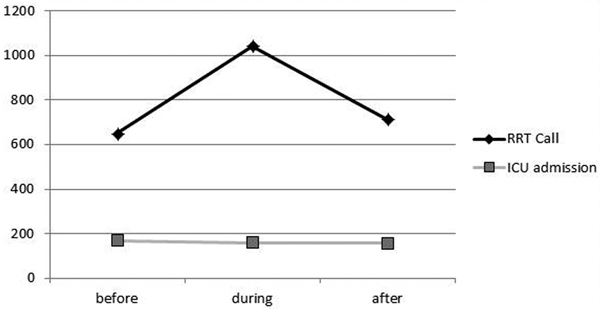
**MET calls and ICU admission before, during and after the RRS implementation**.

**Figure 2:**
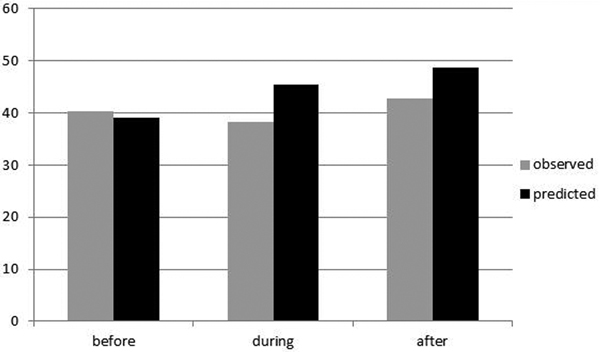
**Hospital mortality predicted and observed before, during and after the RRS implementation**.

The number of MET calls initially increased from 34 to 56 and then decreased to 39 calls/1,000 admissions/year. Most of the calls were from the emergency department and less from medical and surgical wards. The number of ICU admissions did not increase (Figure 1). During the period of study there was a reduction of observed mortality compared to that predicted from SAPS II score, especially in surgical patients (Figure 2). Finally, there was an increase of ICU length of stay (LOS) from 11.5 to 13.7 days and a reduction of hospital LOS from 24 to 23.1 days.

## Conclusion

The implementation of RRS could result in a temporary increase of MET calls but not of ICU admissions; moreover, it could lead to a reduction of mortality and hospital LOS, but not of ICU LOS.
